# Immunological evaluation of patients with 2019 novel coronavirus pneumonia: CD4+ and CD16+ cells may predict severity and prognosis

**DOI:** 10.1371/journal.pone.0268712

**Published:** 2022-08-05

**Authors:** Sam Alahyari, Mohsen Rajaeinejad, Hasan Jalaeikhoo, Leila Chegini, Maryam Almasi Aghdam, Ali Asgari, Malihe Nasiri, Alireza Khoshdel, Ali Faridfar

**Affiliations:** 1 Science and Research branch, AJA University of Medical Sciences, Tehran, Iran; 2 Faculty of Medicine, Shahid Beheshti University of Medical Sciences, Tehran, Iran; 3 AJA Cancer Epidemiology Research and Treatment Center (AJA‐ CERTC), AJA University of Medical Sciences, Tehran, Iran; 4 Resident of Internal Medicine, Faculty of Medicine Aja University of Medical Sciences, Tehran, Iran; 5 Department of Pathology, Shahid Beheshti University of Medical Sciences, Tehran, Iran; 6 Infectious Diseases Research Center, AJA University of Medical Science, Tehran, Iran; 7 Department of biostatics, Shahid Beheshti University of Medical Sciences, Tehran, Iran; 8 Modern Epidemiology Research Center, Aja University of Medical Sciences, Tehran, Iran; LSU Health Shreveport, UNITED STATES

## Abstract

**Purpose:**

Available but insufficient evidence shows that changes may occur in the immune system following coronavirus disease 2019 (COVID-19). The present study aimed at evaluating immunological changes in patients with severe acute respiratory syndrome coronavirus‐2 (SARS-CoV-2) pneumonia compared with the control group.

**Method:**

The present study was performed on 95 patients with COVID-19 (32 severe and 63 moderate cases) and 22 healthy controls. Relationship between immune cells, disease severity and lung involvement was assessed. Binary logistic regression and ROC curve tests were used for statistical analysis.

**Results:**

A significant decrease was observed in CD20^+^ cell counts of the patients. To differentiate patients from healthy individuals, the cutoff point for the CD4^+^ cell count was 688 /μL, sensitivity 0.96, and specificity 0.84. An increase in CD4^+^ cells reduces the odds of severe disease (odds ratio = 0.82, P = 0.047) and death (odds ratio = 0.74, P = 0.029). CD4^+^ cells play a pivotal role in the severity of lung involvement (P = 0.03). In addition to CD4^+^ cells, Fc gamma receptor III (FcγRIII) (CD16) also played a significant prognosis (odds ratio = 0.55, P = 0.047). In severe cases, C-reactive protein, Blood urea nitrogen, and Creatine phosphokinase levels, as well as neutrophil counts, were significantly higher than those of moderate ones whereas lymphocyte count in severe cases was lower than that of moderate ones.

**Conclusion:**

The number of total T-cells and B-cells in patients with COVID-19 was lower than that of controls; however, their NK cells increased. FcγRIII and CD4^+^ cells are of great importance due to their association with COVID-19 prognosis.

## Introduction

Coronavirus disease 2019 (COVID-19) caused by severe acute respiratory syndrome coronavirus 2 (SARS-CoV-2) recently was announced as a global pandemic by the World Health Organization (WHO) on March 11, 2020. The same as MERS-CoV and SARS-CoV, SARS-CoV-2 is from the beta genus Coronavirus in the family of coronaviridae [1]. This can lead to clinical presentations ranging from asymptomatic to mild symptoms like cough, fever, and dyspnea, Acute Respiratory Distress Syndrome (ARDS), and death [2]. Although, the fatality of SARS-CoV-2 is not as high as SARS-CoV-1 or MERS-CoV, noticeable spread of its pandemic has caused lots of devastating consequences for all medical systems and health organizations [3]. Beyond all, human immune responses to the virus remained poorly understood.

COVID-19 may cause lymphopenia [4], but it can also lead to immune hyperresponsiveness called cytokine storm in severe cases [5]. This suggests that in pathogenesis, the host immune system is involved [6, 7]. There are evidences of inflammatory responses, such as rise of IL-6 or GM-CSF-producing CD4T cells or reduced immunoregulatory subsets like regulatory T cells (Treg) or ɣδ T cells [8]. Increase of peripheral T cells’ inhibitory receptor expression or Exhaustion of T cell has been investigated, as well. In addition, T cell activation of COVID-19 patients is reported, although some studies have shown decreasing polyfunctionality or cytotoxicity [9–12]. However, the mechanism of lymphopenia despite the activation of T cells remains unclear in Covid-19 disease [4, 13, 14].

Although, there are many infected people and deaths, the information about the existence and phenotype of SARS-CoV-2-specific T cells is lacking [15]. A recent study announced the SARS-CoV-2-specific T cells presence in convalescent samples of mild COVID-19 patients. They exhibited strong response to viral spike surface glycoprotein (S)-, membrane (M)—and nucleo (N) proteins [16]. Besides, few studies have defined cellular responses in patients. Understanding the pathogenesis of the disease and evaluating the formation of virus-specific CD4 and CD8T cells is of paramount importance due to its contribution to the effective vaccine production process. The aim of this study was to comprehensively evaluate the changes in the immune system in patients with Covid-19 and to compare these changes with healthy individuals.

## Materials and methods

### Sample selection

A total of 95 patients with COVID-19 (aged 21–96 years), admitted to a referral center from 13 June to 01 September 2020, were enrolled in the study. The diagnostic criteria for 2019 novel coronavirus pneumonia (2019-nCoV pneumonia) were: clinical symptoms and positive Real-Time PCR (rtPCR) result. Exclusion criteria were: negative rtPCR result and no chest CT scan findings, or two consecutive negative rtPCR results, and the presence of underlying hematological diseases (e g, acute and chronic leukemia), immunodeficiency, active lymphoma, and undergoing chemotherapy. The study process was explained to the subjects, and written informed consent was obtained. In addition, 22 healthy individuals (aged 23–68 years) with no known underlying disease, referring to the hospital for routine physical examinations with negative PCR results, were selected as the control group in order to compare lymphocyte changes.

Clinical symptoms of patients, including dyspnea, cough, fever, weakness and lethargy, myalgia, nausea and vomiting, and anorexia, as well as vital signs, were recorded. Comorbidities, including diabetes, hypertension, chronic heart disease, and chronic lung disease, were also recorded. Patients were assigned to moderate and severe groups, according to the severity of the disease. Patients were assigned to two groups based on disease severity. Severe and critical cases were considered by the following criteria: fever and respiratory infection plus one of the below-mentioned symptoms of 1- The breath rate more than 30/minute, 2- Severe respiratory distress, 3- blood oxygen saturation less than 93%, 4- lung infiltrates *>*50% of the lung field within 24–48 hours, 5- septic shock, 6- multiple organ dysfunction/failure, 7- respiratory failure [17]. Laboratory tests and radiological examinations were performed on the first day of hospitalization.

### Radiological examination

To evaluate the extent of pulmonary involvement, a chest CT scan without contrast was attempted. Findings on CT images were then interpreted by a qualified radiologist.

Each of the lung lobes was evaluated using a scoring system: 0 = no lobar involvement (0%), 1 = minimal involvement to a lobe (1–25%), 2 = mild lobar involvement (26–50%), 3 = moderate lobar involvement (51–75%) and 4 = severe lobar involvement (76–100%). An overall score was obtained summing the cores of five lobe scores (In the range of 0–20). Severity of lung involvement on CT scan was classified on a 4-point ordinal scale: grade 0 score of 0 (No abnormality present on CT), grade one score of 1–5, grade two score of 6–15 and grade three score of 16–20 [18].

### Laboratory measurements

Upper respiratory throat swab samples were taken from all patients with suspected 2019-nCoV infection on admission and every patient underwent Real-Time PCR with DAAN gene Co. Ltd device. Complete blood cell count, C-reactive protein (CRP), Erythrocyte sedimentation rate (ESR), hepatic aminotransferase and alkaline phosphatase, creatinine, Blood urea nitrogen (BUN), Lactate dehydrogenase (LDH), and creatinine phosphokinase (CPK), were requested. For ruling out other infections, both urine and blood cultures were performed for all the patients. Another blood sample was also taken for flow cytometry at the time of hospital admission. CBC and flow cytometry were also performed on the control group. CD3 (total T cells), CD4, and CD8, CD16, CD20, and CD16/CD56 counts were measured in both the case and control groups.

### Flowcytometry

10 *μ*L of each antibody (CD3-FITC, CD8-FITC, CD16-FITC, Immunostep, Salamanca, Spain) (CD4-PE, CD20-PE, CD56-PE, Beckman-Coulter, Marseille, France) were added to 100 *μ*L whole blood in tubes. Isotype controls included the replacement of specific antibodies with isotype mouse immunoglobulins. After vortexing and incubation for 30 min at room temperature in dark, 2 mL lysing solution was added and RBCs were lysed for 10 min at room temperature. Samples were centrifuged (Hettich Zentrifugen, Switzerland) for 5 min at 460 ×g. After pelleting, 2 mL washing solution was added and samples were centrifuged for 5 min. Prior to analysis, leukocytes were resuspended in 1ml PBS containing 1% formaldehyde. Samples were analyzed using Flow Cytometer device (Partec Cube 6) and *FCS express* Software. Ten thousand events were analyzed per sample. Positive populations were identified using Isotype control.

### Statistical analysis

Data were analyzed using SPSS version 25 (IBM, NY, USA) and GraphPad Prism version 8.0 (GraphPad Software, Inc., San Diego, CA, USA). Descriptive data were expressed as mean and standard deviation. Kolmogorov–Smirnov test was used to test the normality in continuous variables. Independent t-test were used to compare immune cells between the patients and control groups, as well as moderate and severe patients. Spearman correlation was utilized to evaluate the relationship between immune cells and the severity of lung involvement in patients. The Mann-Whitney test was employed to compare laboratory variables between severe and moderate cases. Binary logistic regression model was applied to determine the relationship between immune cells, severity and mortality. Odds ratio (OR) and 95% confidence interval were also reported to show the intensity and direction of the relationship. ROC curve was also plotted to determine the cutoff point for immunologic markers to differentiate the patients from controls. P <0.05 was considered as the level of significance.

### Ethical statement

The study was performed in accordance with Declaration of Helsinki and was approved by AJA University of Medical Sciences ethical committee (number IR.AJAUMS.REC.1399.062).

## Results

A total of 95 patients with COVID-19 (32 severe and 63 moderate cases) and 22 healthy individuals were evaluated in the present study. The mean age of the patients and controls was 63.03±16.75 and 37.04 ± 9.85 years, respectively. There were 56 males and 39 females in the case and 12 males and 10 females in the control groups. In the case group, 21 subjects died, and the rest were discharged. Demographic characteristics of the case and control groups are shown in Table 1.

**Table 1:** Clinical and radiologic features of patients with Covid-19 and control group.

Baseline characteristics	Patients (n = 95)		Control group (n = 22)
	Moderate cases (n = 63)	Severe cases (n = 32)	
Age	62.41±16.14	64.25±17.86	37.40± 9.85
Vital signs			
Blood pressure	125/75	126/74	115/80
Respiratory rate	20±4	25±7	14±2
Heart rate	82±11	93±15	77±9
Temperature	37.2±0.7	37.6±0.8	36.9±0.3
Oxygen saturation	89.4%	87.7%	96%
Primary symptoms			
Dyspnea	42 (67%)	26 (81%)	-
Fever	37 (59%)	19 (59%)	-
Lethargy	38 (60%)	22 (69%)	-
Myalgia	35 (56%)	13 (41%)	-
Nausea and vomiting	14 (22%)	4 (13%)	-
Cough	26 (41%)	11 (34%)	-
Diarrhea	4 (6%)	0 (0%)	-
Anorexia	11 (17%)	5 (16%)	-
Comorbidity			
Chronic heart disease	13 (21%)	8 (25%)	-
Chronic lung disease (asthma and COPD)	5 (8%)	2 (6%)	1
Diabetes	11 (17%)	10 (31%)	2
Hypertension	24 (38%)	8 (25%)	2
Lung involvement grading			
Grade 0	2 (3%)	0 (0%)	-
Grade 1	17 (27%)	4 (13%)	-
Grade 2	38 (60%)	17 (53%)	-
Grade 3	6 (10%)	11 (34%)	-

The most prevalent symptoms of patients on admission were: dyspnea (71%), weakness and lethargy (63%), and fever (58%%). Among the underlying diseases, hypertension had a significant effect on prognosis and the severity of the disease. In addition, the severity of the disease and death rate were significantly higher among the ones with hypertension.

### Laboratory results

Considering the CBC results, 14 patients had mild leukopenia. The WBC count was higher in severe cases than the moderate ones. Also, the neutrophil count was significantly higher in the severe subjects compared to others (P = 0.001). Lymphopenia was observed in 26 (81%) severe and 45 (71%) mild cases; the difference was statistically significant (P = 0.036). Evaluation of other laboratory variables showed that CRP and BUN levels were significantly higher in the severe patients compared to moderate ones (Table 2). The neutrophil count contributes to the prognosis of the disease, so that their number is significantly higher in deceased patients compared with discharged ones (P = 0.029). In addition to the neutrophil count, LDH and CPK also have a significant role on the prognosis of the disease.

**Table 2:** Laboratory variables’ effect on disease severity and prognosis.

	Moderate patients Mean (Std)	Severe patients Mean (Std)	P*	Deceased patients Mean (Std)	Discharged patients Mean (Std)	P*
C-reactive protein (mg/l)	44.37 (34.25)	65.74 (35.33)	0.006	60.94 (37.37)	48.91 (35.74)	0.18
Erythrocyte sedimentation rate (mm/hr)	50.84 (30.39)	46.62 (29.91)	0.53	49.85 (29.96)	49.29 (30.80)	0.925
Aspartate transaminase (U/L)	34.23 (14.60)	37.25 (20.66)	0.47	38.52 (20.97)	34.32 (15.78)	0.348
Alanine transaminase (U/L)	31.71 (13.24)	33.28 (26.35)	0.76	36.42 (30.52)	31.05 (13.94)	0.733
Alkaline phosphatase (U/L)	191.01 (53.67)	223.28 (142.69)	0.23	215.19 (102.46)	198.10 (93.66)	0.563
Creatinine (mg/dl)	1.33 (0.37)	1.43 (0.48)	0.32	1.47 (0.59)	1.34 (0.35)	0.613
Blood urea nitrogen (mg/dl)	21.93 (12.27)	28.84 (12.93)	0.013	29.42 (16.29)	22.79 (11.60)	0.085
Lactate dehydrogenase(U/L)	615.80 (833.27)	682.96 (311.77)	0.66	714.04 (368.04)	616.97 (777.12)	0.010
White blood cells (×10^3^/μL)	6.39 (2.72)	7.61 (4.10)	0.14	7.99 (4.46)	6.46 (2.86)	0.145
Hemoglobin (g/dl)	12.78 (2.49)	12.94 (2.24)	0.77	13.03 (1.66)	12.78 (2.60)	0.784
Platelet (×10^3^/μL)	180.34 (89.33)	187.03 (72.09)	0.72	197.95 (64.25)	178.24 (89.21)	0.097
Lymphocyte count (×10^3^/μL)	1.26 (0.65)	0.96 (0.63)	0.036	1.08 (0.66)	1.19 (0.66)	0.52
Neutrophil count (×10^3^/μL)	4.67 (2.65)	6.17 (3.63)	0.001	6.48 (4.19)	4.81 (2.66)	0.029
Ferritin (μg/L)	481.636(369.26)	602.48 (320.90)	0.18	511.81 (387.33)	522.93 (358.30)	0.847
Creatine phosphokinase (U/L)	200.11(330.98)	386.81 (533.09)	0.03	539.57 (627.90)	184.51 (304.94)	0.001

* Mann-whitney Test.

IQR: Interquartile range.

### Immunologic test results

**Fig 1:**
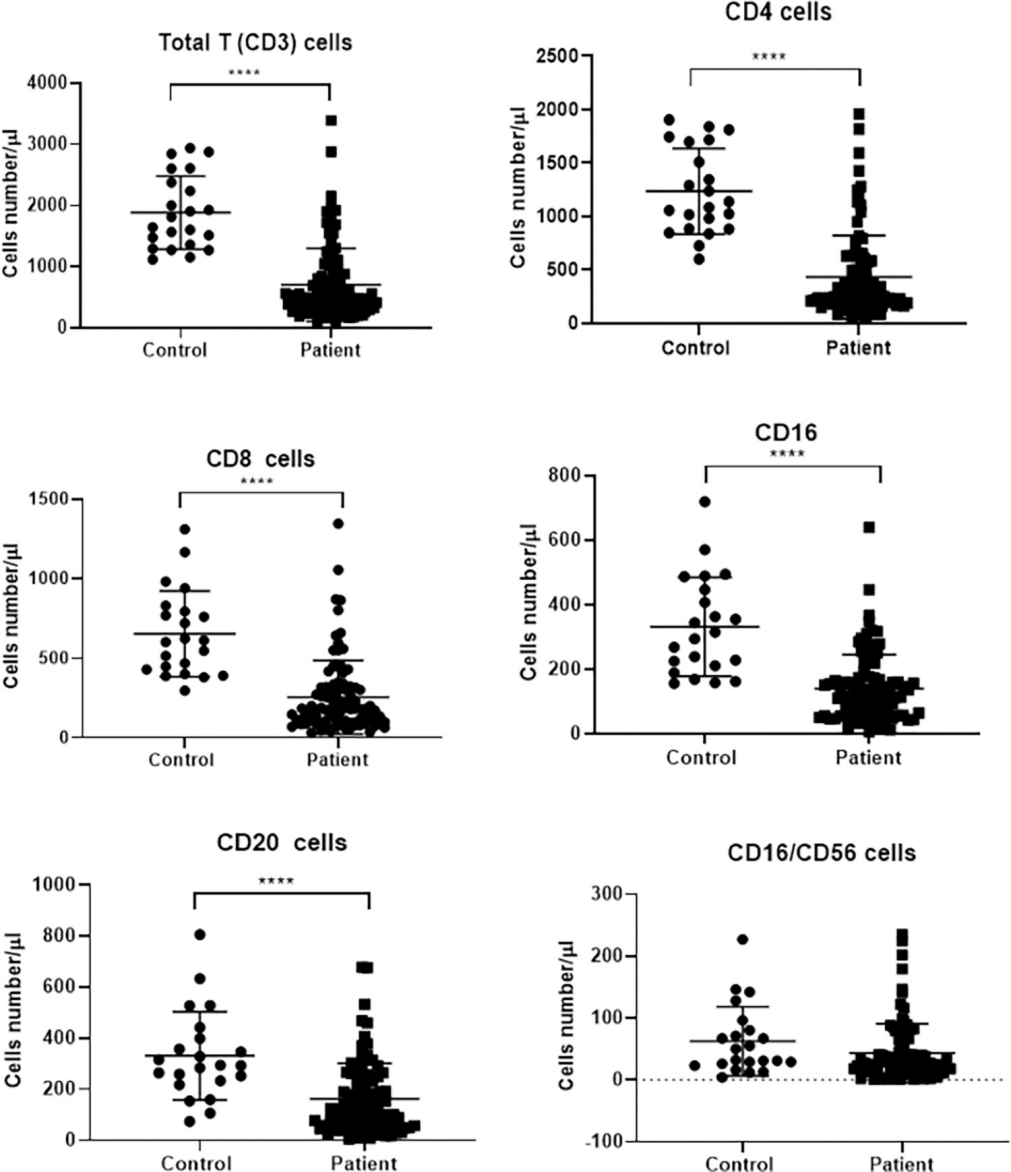
Impact of SARS‐CoV‐2 infection on T, B and NK cell numbers in patients compared with the control group (**** *P< 0*.*0001*). The data are the results of immunological marker analysis in 95 patients and 22 healthy controls. In all studied markers except CD16/CD56, a significant difference was observed between the two groups.

**Fig 2:**
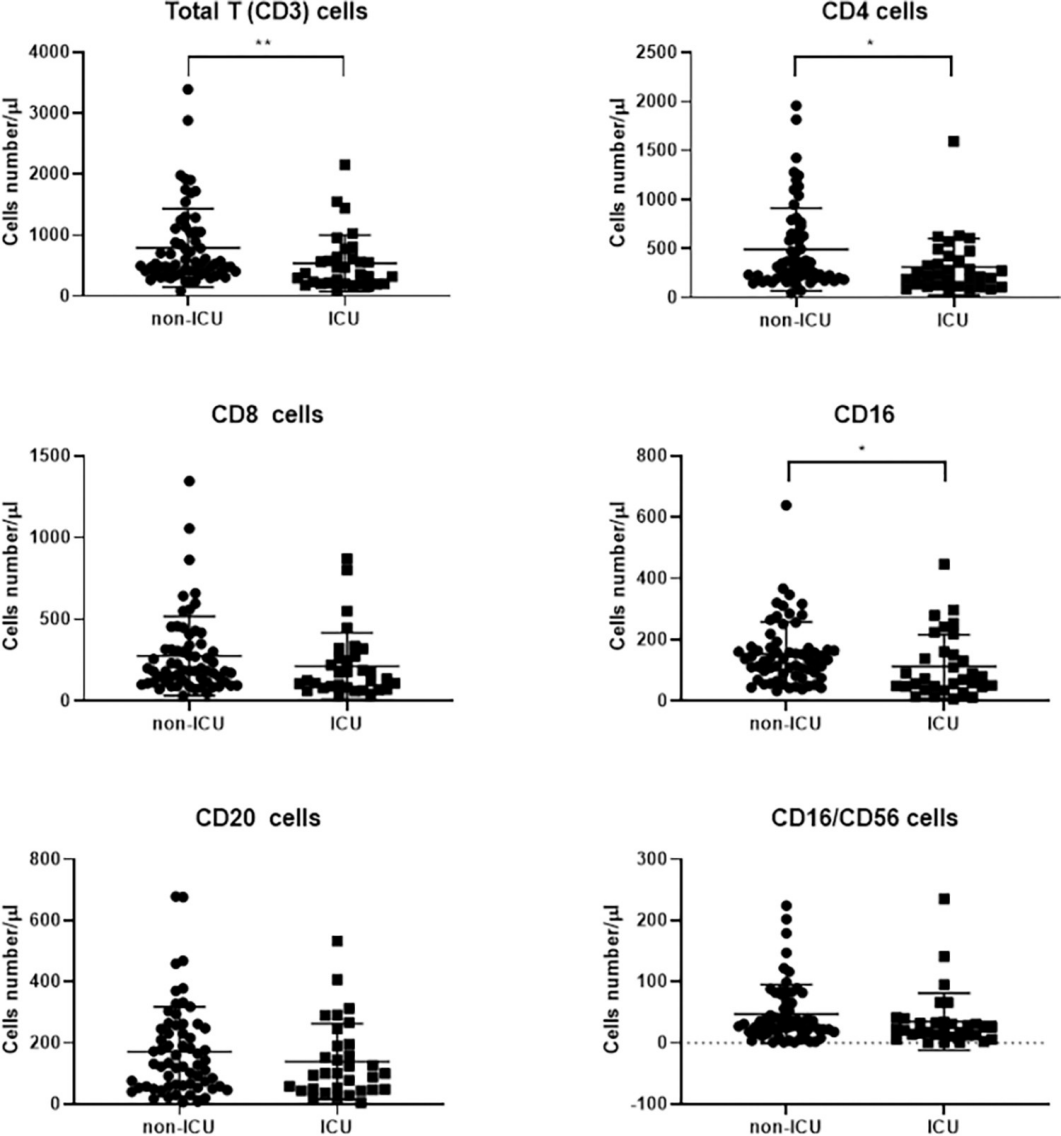
Impact of SARS‐CoV‐2 infection on T, B and NK cell numbers in severe and moderate patients (* P< 0.05, ** P< 0.01). Immunological markers were analyzed in 32 severe cases compared with 63 moderate cases. CD3^+^, CD4^+^ cells and FcγRIII (CD16) had a significant difference between the two groups. Due to the poorer prognosis with an increase in neutrophils that have FcγRIIIB and insignificant changes in NK cell count, the increase in CD16 is due to the increase in peripheral blood monocytes in patients with COVID-19.

Totally, 14 patients had leukopenia (WBC <4000 × 10^9^/L) and 71 lymphopenia. Comparison of the immune systems of the patients and controls immune system showed significant changes in 2019-nCOV pneumonia. CD3^+^, CD4^+^, CD8^+^, and CD20^+^ cell counts had significant differences between the patients and controls (Table 3, Fig 1). However, no significant differences were observed in NK cell count (P = 0.098). In the present study, 32 patients were admitted to ICU. Comparison of the immune cells between the ICU-admitted (severe) and the general ward-hospitalized (moderate) patients indicated significant differences only in CD3^+^ and CD4^+^ cell counts (Fig 2 and Table 3). Differences in other cell counts were insignificant. Also, in moderate cases, the mean Fc gamma receptor III (FcγRIII) (CD16) was much higher than severe cases (P = 0.011).

**Table 3:** Comparison of immune cells in patients (severe and moderate cases) and controls.

Immune cells	Median (/μL) (IQR)		P*	Median (/μL) (IQR)		P*
	Patients	Control		Moderate cases	Severe cases	
CD3^+^	485 (320–882)	1730 (1343–2435)	0.001	522 (389–1054)	368 (202–635)	0.009
CD4^+^	283 (184–575)	1111 (883–1703)	<0.001	330 (200–652)	226 (120–413)	0.016
CD8^+^	182 (100–319)	607 (421–803)	<0.001	184 (116–340)	130 (79–307)	0.115
CD16^+^	113 (56–173)	304 (205–456)	<0.001	135 (82–174)	71 (46–158)	0.011
CD20^+^	123 (54–246)	293 (229–409)	<0.001	132 (58–248)	100 (47–194)	0.244
CD16^+^CD56^+^	28 (16–52)	41 (25–84)	0.06	31 (18–68)	22 (12–33)	0.066

*Independent T-test.

Logistic regression analysis revealed a reduction in the chance of severe disease by 18% for every 100 /μL of increase in CD4^+^ cells in unadjusted model (P = 0.047). Also for every 100 /μL increase in CD3^+^ cells, the chance of severe disease decreases by 10%, this decrease was statistically borderline. (P = 0.06). Although in the adjusted model for age, sex, and comorbidities, odds of developing severe disease for both cells were equal to the unadjusted model, this reduction in the chance of death was statistically borderline. In addition to the cells, it was observed that for every 100 /μL of increase in FcγRIII, the chance of severe disease is reduced by 33% in both adjusted and unadjusted models (Table 4).

**Table 4:** The odds of severity affected by immune cells in patients with COVID-19 per 100.

Immune cells	Model 1*		Model 2**	
	OR (95% CI)	P	OR (95% CI)	P
CD3^+^	0.90 (0.75–1.10)	0.06	0.90 (0.74–1.10)	0.074
CD4^+^	0.82 (0.67–0.99)	0.047	0.82 (0.67–1.00)	0.055
CD8^+^	1.00 (0.99–1.01)	0.99	1.00 (0.99–1.01)	0.99
CD16^+^	0.67 (0.37–1.22)	0.08	0.67 (0.37–1.22)	0.09
CD20^+^	0.82 (0.55–1.22)	0.28	0.87 (0.55–1.15)	0.38
CD16^+^CD56^+^	0.55 (0.20–1.49)	0.24	0.55 (0.20–1.49)	0.26

*unadjusted.

**adjusted for age, sex and comorbidity.

For every 100 /μL of increase in CD4 cells, the chance of death in both adjusted and unadjusted models is significantly reduced. In addition, it was observed that increasing in FcγRIII by 100/μL reduces the chance of death by 50% and 45% in unadjusted and adjusted models, respectively (P = 0.042, P = 0.047 respectively) (Table 5). Due to the lack of increase in the number of NK cells, the increase in FcγRIII could be due to the increase in the number of monocytes in patients with COVID-19.

**Table 5:** The odds of death affected by immune cells in patients with COVID-19.

Immune cells	Model 1		Model 2	
	OR (95% CI)	P	OR (95% CI)	P
CD3^+^	0.90 (0.74–1.00)	0.07	0.90 (0.74–1.10)	0.14
CD4^+^	0.74 (0.61–0.90)	0.029	0.74 (0.61–0.90)	0.029
CD8^+^	1.00 (0.82–1.22)	0.63	0.90 (0.74–1.10)	0.45
CD16^+^	0.50 (0.27–0.90)	0.042	0.55 (0.30–0.99)	0.047
CD20^+^	1.00 (0.67–1.49)	0.81	1.00 (067–1.49)	0.86
CD16^+^CD56^+^	0.5 (0.12–2)	0.32	0.90 (0.15–2.44)	0.14

*unadjusted.

**adjusted for age, sex and comorbidity.

Considering the relationship between the severity of pulmonary involvement in CT images and immune cells, a negative correlation was found between CD3^+^, CD4^+^ and CD16^+^CD56^+^ cell counts and the rate of pulmonary involvement, which was statistically significant.

The ROC curve was plotted for immune cells in order to obtain a cutoff point to differentiate patients from healthy controls (Table 4). In the case of NK cells (CD16^+^CD56^+^), it was not possible to determine the cut-off due to the area under curve below 0.7. Therefore, for absolute CD3^+^, CD4^+^, CD8^+^ and CD20^+^, 1145.5, 688.5, 365 and 104.5/μL were considered as cut-off points, respectively (Table 6). It was not possible to determine cut-off points to differentiate between severe and moderate patients due to the area under curve less than 0.7.

**Table 6:** Determination of lymphocyte cut-offs to differentiate between patients with Covid 19 and healthy controls.

	Cut off (/μL)	Area under curve	P	95% Confidence Interval		Sensitivity %	Specificity %
				Lower Bound	Upper Bound		
CD3^+^ cell counts	1145.5	0.922	<0.001	0.874	0.969	96	84
CD4^+^ cell counts	688.5	0.922	<0.001	0.874	0.970	96	84
CD8^+^ cell counts	365	0.877	<0.001	0.829	0.946	96	80
CD20^+^ cell counts	104.5	0.809	<0.001	0.721	0.898	96	47

## Discussion

COVID-19 pandemic is affecting the world. The number of new cases and victims are increasing daily. COVID-19 can cause a cytokine storm in the patient, resulting in immune dysfunction and death [19]. Therefore, understanding changes in the immune system can promote medical care and reduce mortality. The most common symptoms were dyspnea, weakness, lethargy, and fever. Comparison of laboratory results between severe and moderate cases indicated that the levels of CRP, CPK, and BUN, as well as neutrophil count, were significantly higher in the ICU-admitted patients. Also, lymphocyte depletion rate was significantly higher in severe cases compared to moderate ones. Similar findings were reported in other studies [20, 21]. Some studies reported a significant increase in AST, ALT, and LDH levels in severe cases compared with mild and moderate patients [12]. Such significant changes were not observed in the present study. Patients with hypertension were at risk of poor prognosis and more severe disease. Some studies reported that the highest mortality rate in COVID-19 patients with underlying diseases belonged to the subjects with hypertension [22, 23].

Lymphopenia (lymphocyte count <1500/dL) was detected in 71 patients. In a more detailed study of immune system changes in patients with COVID-19 compared with healthy controls, significant differences were observed. T-cell counts decreased while B-lymphocytes and NK cells increased in the patients. Comparison of immune cells between severe and moderate cases indicated a significant difference in CD3^+^ and CD4^+^ cell counts (P = 0.009, P = 0.016 respectively). However, changes in CD8^+^ and CD20^+^ cell counts were insignificant in severe cases. Zhou et al., evaluating 17 patients with COVID-19, of which five were severe cases, concluded that there was a significant difference in CD4^+^ cell count between severe and mild cases. But no differences were reported in the CD8^+^ cell count [24]. However, Chen et al., evaluating changes in the immune system of 21 patients with COVID-19, of which 10 were severe cases, suggested that CD4^+^ and CD8^+^ cell counts were significantly lower in severe cases than mild ones. B-lymphocyte count in severe cases was also higher than that of moderate ones [12].

CD4^+^ cells are crucial in the acquired immune system. They are involved in the maturation process of B lymphocytes by SLAM-associated protein (SAP) resulting in formation of memory B- and long-lived antibody-producing plasma cells [25–27]. Thus, CD4^+^ cells may play a pivotal role in eliciting broad, long-term antibody responses and protective immunity against most viruses. In addition, these cells are essential for an optimal antibody response against coronavirus infection [28]. Concerning the improved activity of cytotoxic T-cells, the CD40-CD40L interaction is essential in CD4^+^ and Antigen-presenting cells [29]. CD4^+^ cells facilitate the formation of pathogen-specific memory CD8^+^ cells during re-infection [30, 31]. In addition, CD4^+^ cells play an immunomodulatory role by preventing uncontrolled inflammatory responses and helping the generation of memory T-cells [32–34]. Therefore, a decrease in CD4^+^ cell count may cause ineffective immune response and uncontrolled inflammation. The present study found that CD4^+^ cell count significantly reduced in severe COVID-19 cases compared to mild ones, which affects the severity of lung involvement and prognosis of the disease.

The role of neutrophils in the prognosis of respiratory diseases is controversial. Neutrophil infiltration into the inflamed lung is a hallmark of Acute respiratory distress syndrome (ARDS) [35]. Besides, activated neutrophils cause oxidative stress, leading to damage to the lungs by secreting proteases and creating neutrophil extracellular traps [36]. However, based on current evidence, neutropenia does not result in improved recovery in ARDS cases [37]. In the present study, an increase of neutrophil count in the peripheral blood led to increased disease severity and poor prognosis in the patients. Similar results are reported in other studies [38].

The present study found that FcγRIII (CD16), which has two isoforms, plays an important role in disease severity and prognosis. There are two types of FcγRIII. FcγRIIIA (CD16A) is an FcγR-associated transmembrane receptor expressed by NK cells, NKT cells and monocytes [39–43]. FcγRIIIB (CD16B) is a GPI-anchored receptor with no intracytoplasmic domain [44]. FcγRIIIB is expressed only in human polymorphonuclear neutrophils [45]. A variety of cytokines including IFN-γ are released when FcγRIII is activated by antigen-antibody complex that signal to other immune cells. The secretion of cytotoxic mediators such as perforin and granzyme causes apoptosis in the target cell. This process is known as antibody-dependent cell-mediated cytotoxicity (ADCC).A recent study found that, despite the step-by-step generation of specific antibodies against the virus in patients with COVID-19, there are two disadvantages to these antibodies: 1- They have a low affinity with Fc receptors and cannot activate the ADCC mechanism properly. 2- They activate the complement cascade, leading to leukocytes invasion and inflammation, which results in tissue damage [46]. In addition, some studies have reported other evidence of immune dysregulation in the form of autoantibodies in particular antinuclear antibody (ANA) which is significantly related to severe lung disease [47]. Among the different ANA patterns, nucleolar ANA reactivity found more commonly which can be the serological marker of systemic sclerosis. It is important to note that among the clinical manifestations of systemic sclerosis, there is pulmonary involvement in the form of a restrictive syndrome secondary to interstitial pneumopathy similar to COVID-19 interstitial pneumonia [48, 49]. Occurrence of other types of autoantibodies such as anti-platelet autoantibodies was reported [50, 51]. On the other hand, opsonizing antibodies against COVID-19 are found in the plasma of recovered patients that can neutralize the virus by the ADCC mechanism. These antibodies show more affinities with FcγRIII (CD16) [46]. In an in-vivo study, DiLillo et al., showed that the stalk-specific antibodies need interaction with FcγRs for protection against influenza virus, which is intensified in case of increased affinity with FcγRIIa and FcγRIIIa [52]. Due to the poorer prognosis with an elevated neutrophil count, the increase in CD16 in recovered patients is attributable to monocytes and NK cells. Due to the lack of significant differences in CD56^+^ and CD16^+^CD56^+^ cells between severe and moderate patients as well as deceased and discharged patients, it seems that the increase in FcγRIII can be due to the increase in peripheral blood monocytes in patients with COVID-19. CD16^+^ monocytes are divided into two subgroups: intermediate (CD14^+^, CD16^+^) and non-classical (CD14^+^, CD16^++^) monocytes [53]. Intermediate monocytes comprise about 2–8% of circulating monocytes. Their functions include production of reactive oxygen species (ROS), antigen presentation, participating in the proliferation and stimulation of T cells, inflammatory responses, and angiogenesis. Non-classical monocytes comprise about 2–11% of circulating monocytes. They can have pro-inflammatory behavior and secrete inflammatory cytokines in response to infection. These cells are also involved in antigen presentation and T cell stimulation [54].

A major limitation of the present study was the lack of flow cytometry repeating in patients recovered from COVID-19 in order to make a pre- and post-treatment comparison. Another limitation of this study was the lack of measurement of monocyte immunologic markers to more accurately assess their role in prognosis and disease severity. Also, conducting this study with a larger number of patients can lead to a more accurate assessment of changes in immune cells in COVID-19.

In conclusion, the present study found significant differences in immune cell counts between patients with COVID-19 and healthy individuals. This difference was observed in the CD3^+^, CD4^+^, CD20^+^, and NK cell counts. Besides, it was found that CD4^+^ cells play a pivotal role in the disease process. These cells had a significant effect on the prognosis and severity of lung involvement in COVID-19. Also, CD4^+^ count in severe cases was significantly lower than that of moderate ones. In addition, increasing the amount of FcγRIII reduced the chances of severe disease and mortality and this increase in FcγRIII was attributable to monocytes. Finally, comparison of laboratory results showed that CRP, CPK, and BUN levels, as well as neutrophil count, were higher in severe cases than moderate ones; they also had lower circulating lymphocytes than moderate patients.
